# Comparative Genomic and Phylogenetic Approaches to Characterize the Role of Genetic Recombination in Mycobacterial Evolution

**DOI:** 10.1371/journal.pone.0050070

**Published:** 2012-11-26

**Authors:** Silvia E. Smith, Patrice Showers-Corneli, Caitlin N. Dardenne, Henry H. Harpending, Darren P. Martin, Robert G. Beiko

**Affiliations:** 1 School of Medicine, Department of Ophthalmology and Visual Sciences, University of Utah, Salt Lake City, Utah, United States of America; 2 Department of Anthropology, University of Utah, Salt Lake City, Utah, United States of America; 3 Department of Biology, Univerity of Utah, Salt Lake City, Utah, United States of America; 4 Computational Biology Group, Institute of Infectious Diseases and Molecular Medicine, University of Cape Town, South Africa; 5 Faculty of Computer Science, Dalhousie University, Halifax, Nova Scotia, Canada; Georgia Institute of Technology, United States of America

## Abstract

The genus *Mycobacterium* encompasses over one hundred named species of environmental and pathogenic organisms, including the causative agents of devastating human diseases such as tuberculosis and leprosy. The success of these human pathogens is due in part to their ability to rapidly adapt to their changing environment and host. Recombination is the fastest way for bacterial genomes to acquire genetic material, but conflicting results about the extent of recombination in the genus *Mycobacterium* have been reported. We examined a data set comprising 18 distinct strains from 13 named species for evidence of recombination. Genomic regions common to all strains (accounting for 10% to 22% of the full genomes of all examined species) were aligned and concatenated in the chromosomal order of one mycobacterial reference species. The concatenated sequence was screened for evidence of recombination using a variety of statistical methods, with each proposed event evaluated by comparing maximum-likelihood phylogenies of the recombinant section with the non-recombinant portion of the dataset. Incongruent phylogenies were identified by comparing the site-wise log-likelihoods of each tree using multiple tests. We also used a phylogenomic approach to identify genes that may have been acquired through horizontal transfer from non-mycobacterial sources. The most frequent associated lineages (and potential gene transfer partners) in the *Mycobacterium* lineage-restricted gene trees are other members of suborder Corynebacterinae, but more-distant partners were identified as well. In two examined cases of potentially frequent and habitat-directed transfer (*M. abscessus* to *Segniliparus* and *M. smegmatis* to *Streptomyces*), observed sequence distances were small and consistent with a hypothesis of transfer, while in a third case (*M. vanbaalenii* to *Streptomyces*) distances were larger. The analyses described here indicate that whereas evidence of recombination in core regions within the genus is relatively sparse, the acquisition of genes from non-mycobacterial lineages is a significant feature of mycobacterial evolution.

## Introduction

Assessing how mycobacteria acquire and maintain the genetic variation needed to both adapt to host immunity and to evolve drug resistance is of paramount importance in efforts to eradicate diseases such as tuberculosis and leprosy. Until recently, inheritance in mycobacteria was believed to be mostly vertical, partially because within individual lineages, bacteria displayed a generally low degree of genetic diversity. While larger genomic surveys have directly challenged this supposition (e.g. [1], [2]), other factors such as the isolation of mycobacterial niches within host cells, slow replication rates, prolonged latent stages, and small effective population sizes, may also explain the low degrees of genetic diversity observed within mycobacterial species [3].

The generation and maintenance of genetic variation plays an essential role in the evolution of all life forms. Genetic diversity, the raw material upon which natural selection operates, is especially important in species inhabiting rapidly changing environments as it ensures the existence of genetic variants with potential adaptations to a wide range of possible growth conditions. It is very important, however, that following an environmental change and the subsequent outgrowth of individuals carrying the appropriate adaptive mutations, mechanisms exist whereby population-wide genetic diversity is either preserved or is rapidly reestablished before the next environmental shift occurs. Whereas, for example, high mutation rates can ensure the reestablishment of novel genetic variation, high rates of genetic recombination coupled with selection can permit the spread of adaptive mutations throughout a population and, in so doing, preserve population-wide genetic diversity following environmental changes [4]–[7].

Recombination is an essential step in the acquisition of DNA by a recipient organism: once donor DNA has entered the recipient cell, it must recombine into the host genome in order to be inherited (except in the case of self-replicating plasmids). Recombination in bacteria can proceed through two primary mechanisms, each represented by a number of different pathways. Homologous recombination involves the replacement of genome regions in a recipient bacterium by homologous sequences from a donor, with strong dependence on sequence identity between the recombining sequences and a sharp drop-off in efficiency with increasing sequence divergence [8], [9]. Non-homologous recombination involves the introduction to a recipient genome of donor-derived genome fragments without the same requirement for sequence similarity and does not involve the replacement of an existing gene within the recipient genome (i.e. the recipient genome increases in size). In this work, we are interested in the products of both types of recombination when they enable the acquisition of genetic material via horizontal gene transfer (HGT), so in most cases we do not distinguish between the two processes.

HGT is known to have occurred in many other genera within the phylum Actinobacteria [10]–[13]. HGT in mycobacteria is plausible since infections by multiple mycobacterial strains have been documented, which can enable HGT due to close proximity of donor and recipient taxa 14,15; additionally, mycobacteria have plasmids, which facilitate HGT events in other microorganisms [16], [17]. Various studies have reported the occurrence of recombination events in multiple mycobacterial species and individual genes, although the mechanisms by which these reported events might have occurred are still unclear. Published studies can be grouped into different categories depending on the methodology used to assess the occurrence of HGT.

The simplest approach to test for the occurrence of HGT is the comparison of either genomic sequences or gene products from different bacterial species. In some cases, the unique presence of a group of genes in one mycobacterial species is used as the starting point for further analyses, which in turn can include G+C content comparison of the unique genes with their flanking regions [18], and/or the characterization of a different class of enzymes [19] or protein subfamily [20] being encoded by the novel genes. Others have proposed the occurrence of HGT upon finding mobile and insertion elements, and/or genomic islands [21]. Another indirect line of evidence is represented by the presence of repetitive dispersed elements, which result from gene duplication or from mobile genetic elements that have multiplied. For example, the transposases of *M. tuberculosis* insertion elements (IS) IS*1552*, IS*1557*, and IS*1561* show remarkable similarities to the IS elements found in *Rhodococcus opacus*, *Terrabacter sp.*, and *Nocardia asteroides* [22]. *M. tuberculosis* may have acquired these IS elements by HGT prior to its niche change from a soil saprophyte to an obligate pathogen [23]. IS*6110*, which was initially thought to be restricted to the *M. tuberculosis* complex, has also been identified in *M. smegmatis* [24].

Wu and collaborators [25] found 24 genomic islands comprising 846 kb of sequence that is present in *M. avium* strain *avium,* but which is absent in 95% of the 34 *M. avium paratuberculosis* strains examined. Some of the genomic islands, whose functions varied, contained mobile elements suggesting that non-homologous recombination events may have led to their insertion or deletion. This is further supported by the presence of enzymes such as transposases and integrases that enable HGT events. Other studies have demonstrated the existence of a conjugation or transduction system in the genus. Conjugation and transduction are known mechanisms of HGT in bacteria, which sometimes rely on plasmids and phages to transfer genetic material from one bacterium to another [26], [27]. Over 155 mycobacteriophage genome sequences have been sequenced to date [28]–[30]. The fact that these mycobacteriophages form numerous phylogenetic clusters, subclusters, and singletons, indicates that the total range of mycobacteriophage genetic variation has not been thoroughly sampled. Comparative analysis of their genomic sequences showed a high level of mosaicism, which may be largely due to HGT [28]–[30].

Given the abundance of data suggesting that HGT could in fact play an important role in mycobacterial evolution, we tested the null hypothesis of mycobacterial clonality using a data set comprising 2354 homologous mycobacterial genome sections representing 18 strains consisting of 13 species. Using phylogenetic approaches, we also attempted to quantify the extent to which members of genus *Mycobacterium* have shared genes with other lineages. Different mycobacteria occupy a wide diversity of habitats including many different host body sites such as skin and lung. As a consequence, we can examine the extent to which mycobacterial genes show phylogenetic cohesion to each other, versus the potential influence of HGT from other microorganisms in similar habitats. Whereas we find only sporadic evidence of recombination between homologous “core” genomic regions within the genus, our analysis of genomic content indicates that the acquisition of novel genes via HGT has been a significant feature of mycobacterial evolution. At least two mycobacterial named species, *M. abscessus* and *M. smegmatis*, have many genes with stronger affinities to non-mycobacterial partners than to other members of the same genus. These putatively transferred genes are involved in a small number of biochemical functions with strong relevance to the microorganisms’ main habitat.

## Results

### Reference Trees of Mycobacterial Genomes

**Figure 1:**
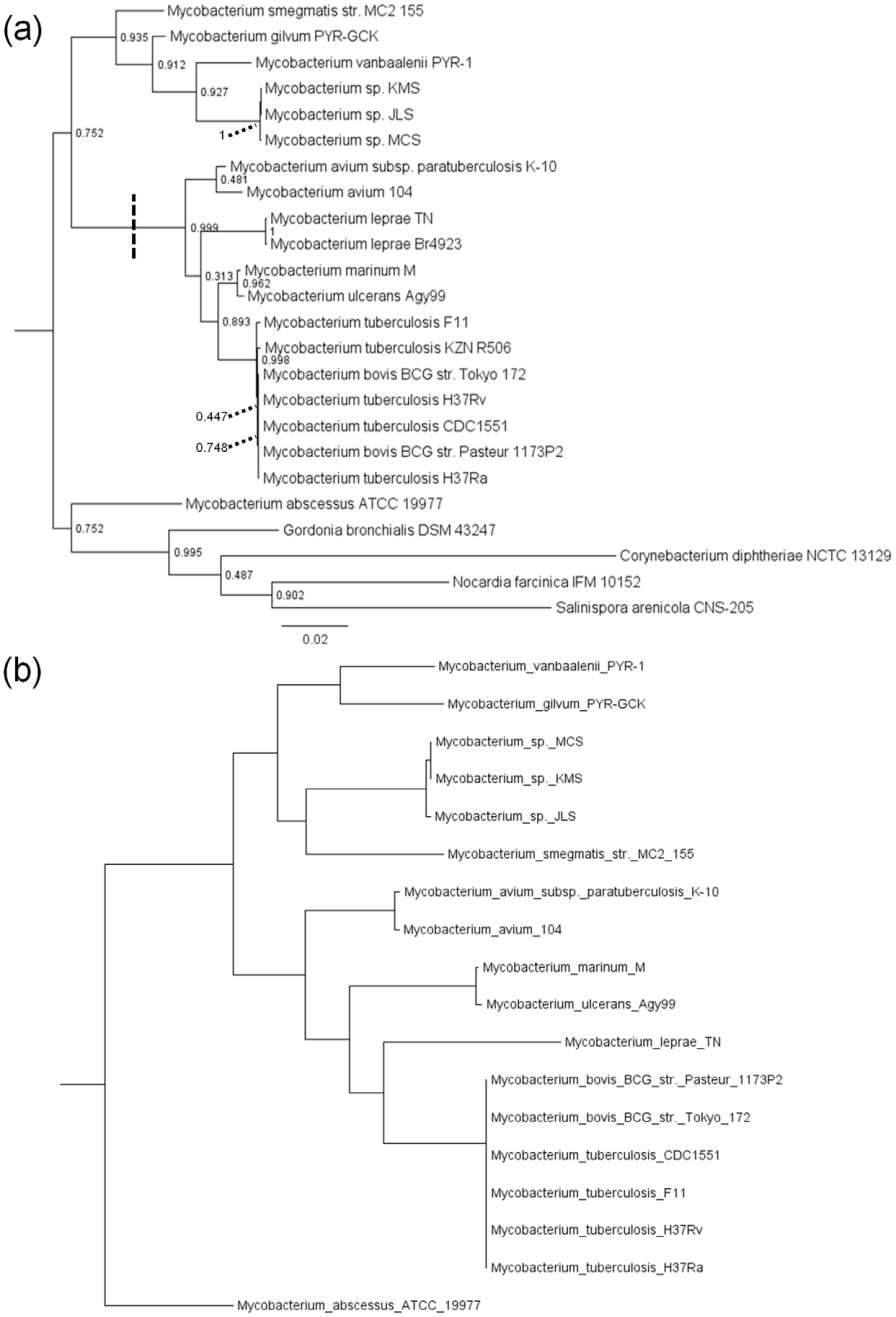
Trees of genus *Mycobacterium* based on 16S rRNA gene sequences (a) and concatenated core regions (b). Trees were inferred using FastTree (a) and concatenated core regions of the genome excluding all recombination events, inferred using RaxML (b). Internal node labels in (a) indicate SH-based “local support” values, and the division between “slow growing” and fast growing” mycobacteria is indicated with a dashed line. All bootstrap support values in (b) were either 99% or 100%, and are consequently not shown.

A total of 20 genomes from genus *Mycobacterium* were used in this study: the names, accession numbers, and identified habitats of these are shown in Table S1. We first assembled a canonical reference topology for the 20 genomes used in this study (18 in the homologous recombination analysis, plus an additional two in the phylogenomic study) by extracting the small-subunit 16S rRNA genes and building a phylogenetic tree. 16S rRNA gene sequences from four other members of suborder Corynebacterinae were included to root the mycobacterial tree (Figure 1a). Most groupings of interest in the tree are well supported, with bootstrap values >0.9 for the entire genus, the *M. tuberculosis* complex (which also includes *M. bovis* and other species not included in this study), *M. leprae*, and many implied clades containing multiple named species. Sequences were typically identical within named species, with one exception in *M. tuberculosis*, while the maximum distance between any pair of mycobacterial 16S sequences was 0.06 substitutions/site. The smallest distance between a mycobacterial 16S sequence and a sequence from another member of Corynebacterinae was 0.0589 substitutions/site, between *M. gilvum* (the shortest branch in Figure 1a) and *Gordonia*. Where relationships can be evaluated, our tree is also consistent with that of van Pittius et al. [31]. Notably, the separation between “slow growers” and “fast growers” is readily apparent: all “fast grower” taxa have genomes in excess of 5.9 Mb, with the exception of the earliest branching organism, *M. abscessus*. The “slow grower” genomes are more heterogeneous in size, with *M. marinum* and *M. ulcerans* in excess of 5.5 Mb, the *M. tuberculosis* complex approximately 4.4 Mb, and the reduced *M. leprae* genomes ∼3.2 Mb. The pairing of the two strains of *M. avium* is poorly supported, leaving open the possibility that *M. avium* is a paraphyletic named species. Larger trees built with more sequences from sources such as GreenGenes [32] indicate greater intermingling of the species included in this analysis, and the tree proposed here is not representative of the phylogenetic cohesion of all isolates that have been assigned to these named species. Nonetheless, the tree provides a useful scaffold for the examination of implied recombination events and phylogenetic relationships of protein-coding genes within the group.

To assess whether genomic relationships are consistent with those suggested by marker genes, we concatenated all readily detectable homologous segments that were found in all 18 of the analyzed mycobacterial genomes into a single 720,090 nt alignment. The tree constructed from this alignment is shown in Figure 1b. Some differences are observed with respect to the 16S rRNA gene tree: for example, the tree from the concatenated alignment supports *M. leprae* as sister to the *M. tuberculosis* complex instead of the *M. marinum*/*M. ulcerans* pairing, and there are differences in the branching order of the “fast-growing” group. However, this tree does support the pairing of *M. avium* strains, and separates the two named species in the *M. tuberculosis* group. In additional analyses we considered the relationships among genes found in all genomes in the data set (core genes), and the phylogenetic affinities of genes that were found in one or more named mycobacterial species.

### Homologous Recombination in Core Regions

We screened the concatenated alignment of conserved homologous segments for evidence of recombination events using RDP version 3.42 [33]. RDP identified a total of 74 potential recombination events. However, as shown in Table S2, only 28 of these were retained at the chosen threshold of statistical significance (multiple-test corrected α ≤0.05) by three or more different methods implemented in RDP3.42 [33]. For each of these 28 putative homologous recombination events, we sought independent phylogenetic verification of the detected recombination signals via maximum-likelihood tree construction and statistical tree comparison tests (see Methods). These tests were used to assess the significance of topological differences between the trees constructed from each recombinant section alone and the tree computed using the non-recombinant portion of the *Mycobacterium* full genome alignment. If the ML tree constructed from the recombinant portion better reflected the relationships of the sequences in the genomic region between the inferred recombination breakpoints, this was interpreted as strong phylogenetic support for the hypothesis that recombination had occurred.

As is shown in Table S2, the null hypothesis (i.e. that the detected signals of recombination were not phylogenetically supported) could clearly not be rejected for 8/28 of the recombination events detected by RDP3.42 (RDP events 11, 12, 22, 24, 31, 45, 48, and 49). Furthermore the null hypothesis could not be convincingly rejected for another 11/28 of the recombination events. Specifically, whereas events 27 and 39 yielded only marginally significant evidence that the tree topologies obtained using the recombinant fragments fitted the data significantly better than the tree topology determined from the 720,090 nt long original alignment (*i.e.,* yielded test p-values between 0.05 and 0.01), events 7 and 10 only yielded statistically significant p-values for the AU test, events 8, 13, 16, 28, 33, 34, and 74 only yielded significant p-values for the AU and WSH tests and none of these yielded significant p-values with the SH test.

Only 9/28 of the detected events (events 1–6, 9, 19, and 37) were supported by all three tests. Since the SH test tends to get more conservative as the number of trees increases [34], it is perhaps expected that it should be the most conservative of the three tests performed here. The AU test [34] was designed to compensate for the SH test’s selection bias, using a multiscale bootstrap, and is thus a reasonable statistic to use for our dataset. On the other hand, the WSH test is less conservative than both the SH and the AU test, and was devised specifically to compensate for the excessively conservative nature of the SH test. It is important to note that both the SH and WSH tests rely on the assumption that the true ML tree is among the trees tested [35]. Thus, depending on the test statistic chosen, we have phylogenetic support for either 19 (the number of events with significant p-values for at least the AU test or the SH test), 13 (the number of events with significant p-values for the AU and the WSH tests), or 9 recombination events (the number of events for which all three tests yielded p-values <0.05). Consequently within the mostly highly conserved core regions of mycobacterial genomes, we were able to detect statistically supported evidence of at least nine different homologous recombination events. All of these potential recombination events, including the recombinant sequence(s) and potential parental sequence(s) are described in detail in Table S3.

### Phylogenetic Affinities of Mycobacterial Genes

The recombination analysis above targeted “core” genomic regions common to the set of sequenced mycobacteria, but a more-complete picture of mycobacterial evolution requires the consideration of variable and lineage-restricted genes as well. To this end, we performed a phylogenomic analysis of predicted mycobacterial proteins to identify candidate gene-sharing partners that are found frequently in association with one or more named species of *Mycobacterium.* We focused on sequences from the NCBI database of 16,118,048 non-redundant protein sequences that are similar to the query protein (*i.e*., BLAST bitscores within 75% of the top BLAST match outside the originating species), since more-distantly related proteins are unlikely to represent recent acquisitions by or donations from the group via HGT. Homologous sets for each protein from each of the 20 mycobacterial genomes in our analysis were generated in this fashion, with interesting subsets subjected to phylogenetic analysis.

Mycobacterial proteins were assigned to one of four groups, based on their phylogenetic distribution. Group I consisted of proteins with no matches (*i.e*., orphan proteins), or whose close matches were restricted to other members of genus *Mycobacterium*. Group II consisted of proteins with matches restricted to *Mycobacterium* and other genera in the suborder Corynebacterineae. Proteins in Group III had matches potentially to *Mycobacterium* but to no other members of Corynebacterineae, and had matches outside this suborder, i.e. to other groups of Actinobacteria, other bacterial phyla, or to archaea and eukaryotes. Finally, Group IV proteins had matches both to members of Corynebacterineae, and to organisms not in this group. Although any protein in Groups II-IV could potentially show phylogenetic evidence of HGT if the full set of mycobacterial sequences were not recovered as a clan, Group III proteins are particularly interesting because they lack matches to the closest taxonomic relatives of *Mycobacterium*, but have matches to more-distantly related organisms.

**Figure 2:**
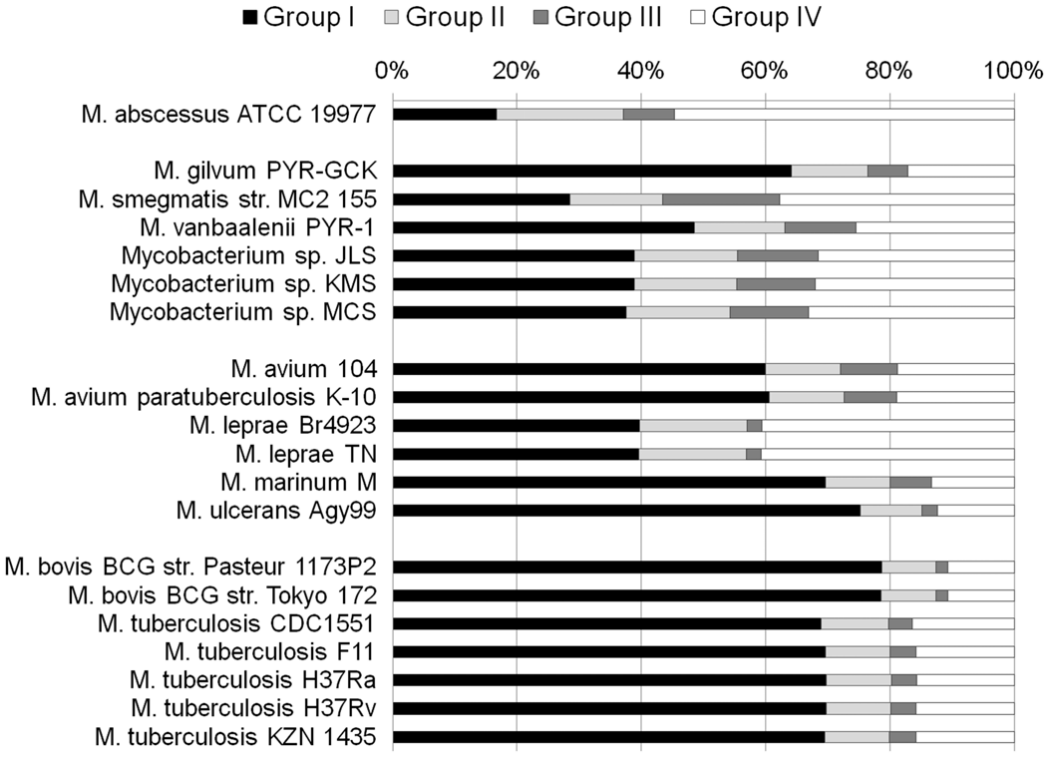
Percentage of protein sets from each mycobacterial strain assigned to Groups I-IV. Strains are grouped according to the four main divisions identified in the text.

Figure 2 shows the distribution of assignments to these four classes for each of the twenty mycobacterial genomes considered. Affinity patterns were similar among strains of the same named species (*i.e*., *M. avium*, *M. bovis*, *M. leprae*, and *M. tuberculosis*). The fraction of proteins assigned to Groups I and IV yielded linear regression coefficients between 0.21 and 0.28 when compared with the minimum 16S rRNA gene distance to any other sequence in the set of 20 genomes. Group I correlated negatively with increasing 16S rRNA gene distance: the earliest-branching member of the group, *M. abscessus*, had the smallest proportion of Group I proteins. More broadly distributed proteins (Groups II and IV) showed positive correlations with 16S rRNA gene distance: *M. abscessus* had the highest proportion of Group II and IV assignments, and *M. bovis* the lowest. Group III proteins, with their unusual phylogenetic distribution, showed a weaker relationship with 16S rRNA gene distance (R^2^ = 0.15). Group III proteins distinguish the otherwise similar *M. marinum*/*M. ulcerans* pairing, and are proportionally fewer in *M. abscessus* and *M. gilvum* than the other “fast-growing” mycobacteria, while *M. smegmatis* has the highest proportion of Group III proteins (18%).

**Figure 3:**
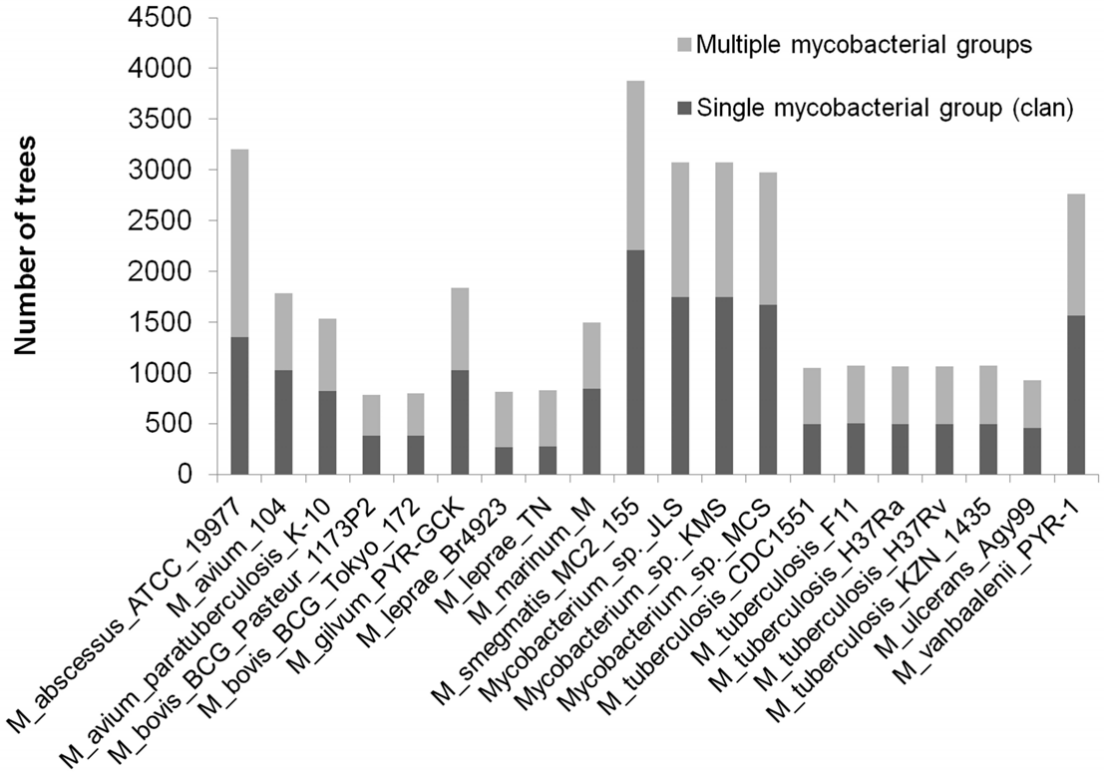
Recovery of cohesive mycobacterial clans. The number of trees for proteins in Groups II, III and IV from each mycobacterial genome that recovered genus *Mycobacterium* as a clan (dark grey) or as multiple groups interspersed with proteins from other genera (light grey) is shown.

We next examined the tendency of mycobacterial gene trees to recover all represented members of the genus as an unrooted group (i.e., a *clan*: [36]). Although recovery of genus *Mycobacterium* as a clan does not rule out the possibility of transfer within the genus, it would suggest a lack of transfers between members of this genus and other organisms. Surprisingly, for many members of the group fewer than half of all gene trees resolve genus *Mycobacterium* as a clan (Figure 3). The majority of organisms that disrupt trees of mycobacterial proteins are other Actinobacteria, and in particular other members of the Corynebacterinae. *Gordonia*, *Nocardia* and *Rhodococcus* each appear in over half of the disrupted mycobacterial clans, suggesting relatively short-distance transfers of genetic material either into or out of mycobacterial species. Considering the *M. bovis* complex alone, the majority (∼90%) of inferred trees recovered a clan containing *M. bovis* and *M. tuberculosis*. Of the approximately 10% of trees in which members of these two named species were split, all but twelve had the *M. bovis*/*M. tuberculosis* clan disrupted only by other mycobacteria. All but one of the remaining twelve trees had an intervening group that comprised *Mycobacterium* and other genera, usually from the same suborder. The last tree had *Rhodococcus equi* as the lone intervening group. The corresponding gene, annotated as translation initiation factor IF-1, is a clear example of a transferred gene as the copies found in *M. tuberculosis* and *R. equi* are identical.

**Figure 4:**
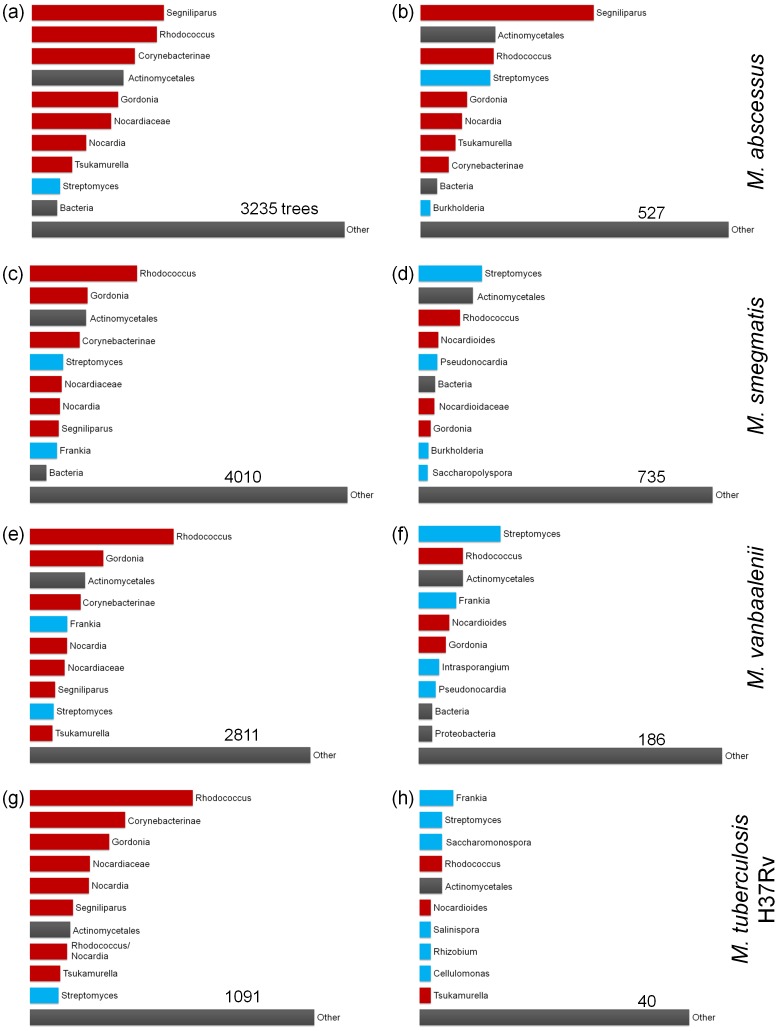
Closest-neighbor analysis of trees of proteins from four mycobacterial genomes. Each row corresponds to a different genome. The left column (a, c, e, g) summarizes the taxonomic composition of the most taxonomically limited neighboring group for all trees containing proteins from a particular organism, while the right-hand column shows the same information for trees containing proteins whose distribution among the mycobacteria is limited to the target species (or complex, in the case of *M. tuberculosis*). Red bars indicate taxonomic groups that are completely contained within suborder Corynebacterinae, blue bars show groups that have no overlap with Corynebacterinae, and grey bars indicate groups that contain both Corynebacterinae and non-Corynebacterinae.

The nearest neighbors of named mycobacterial species or complexes can correspond to either donor or recipient partners with these lineages. Systematic profiling of partners revealed considerable differences among the mycobacteria: Figure 4 summarizes the ten most-frequent partner lineages of mycobacterial groups containing *M. abscessus*, *M. smegmatis*, *M. vanbaalenii* and *M. tuberculosis*, for all trees (Figure 4 a, c, e, and g) and for sets of trees in which the mycobacterial species or complex is the lone representative of the genus (Figure 4 b, d, f, and h). *M. abscessus* has previously been found to contain many virulence genes putatively acquired from other organisms within Corynebacterinae (e.g., *Rhodococcus*), from non-Corynebacterinae Actinobacteria (e.g., *Streptomyces*), and from other phyla (e.g., *Pseudomonas* and *Burkholderia*) [37]. We observed strong affinities with other Corynebacterinae when all other gene trees were considered (Figure 4a), and *Segniliparus*, *Rhodococcus,* and *Streptomyces* as the most-frequent partners of genes found in *M. abscessus* and no other mycobacteria. The genome sequence of *Segniliparus rotundus* was not available in the previous study, having only been published in 2010 [38]. Both *M. abscessus* and *S. rotundus* are pathogens associated with lung disorders [39], and are thus particularly good candidates for ecology-driven gene transfer. Lineage-restricted genes of *M. smegmatis* and *M. vanbaalenii* show similar profiles, with *Streptomyces* and *Rhodococcus* as the most-frequent putative donors. *M. vanbaalenii*, however, has far fewer lineage-restricted genes relative to its genome size, possibly reflecting its closer proximity to other lineages in the tree of sequenced mycobacterial genomes (Figure 1a). The affinities of *M. tuberculosis* H37Rv are dominated by other Corynebacterinae, with only 40 genes restricted to the *M. tuberculosis* complex. No partner lineage dominates among these 40 genes: *Frankia* is the nearest neighbor in three trees, three genera are partners exactly twice, and the remaining partners are observed only once.

**Figure 5:**
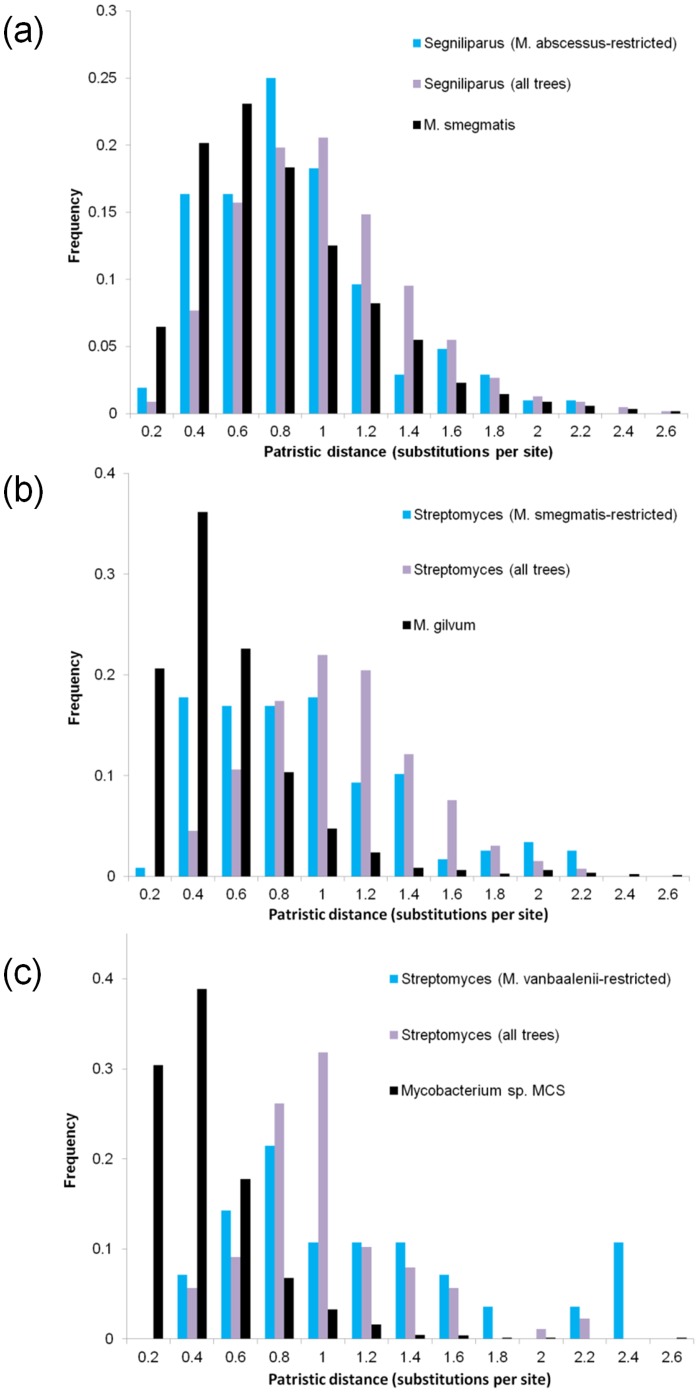
Patristic distances for genes from selected pairs of genomes. Distribution of patristic distances (in substitutions per site) between protein sequences from ‘target’ mycobacteria (a, *M. abscessus*; b, *M. smegmatis*; c, *M. vanbaalenii*), non-mycobacterial genera with strong affinities to the target mycobacterium, and ‘calibrating’, closely related mycobacteria. Blue bars show the distribution of distances from sequences found in the target species and no other mycobacteria to the non-mycobacterial group; purple bars show a similar distribution for sequences present in other mycobacterial as well; and black bars show the distribution of distances between the target and calibrating mycobacterial genomes.

Genes that are rare in *Mycobacterium* but have similarity to other taxonomic groups could have been present in the ancestral population of all mycobacteria and lost in most descendant lineages, or acquired from the other group through gene transfer. Vertical inheritance coupled with loss implies a much earlier divergence time and more sequence change between the mycobacterial copies and the copies present in the external lineage, whereas recent HGT events will produce more-similar genes in the two lineages. For the three mycobacterial species above that showed affinities to other lineages (*M. abscessus* to *Segniliparus*, and *M. smegmatis* and *M. vanbaalenii* to *Streptomyces*), we examined the distribution of patristic distances (*i.e*., the sum of branch lengths in a gene tree) for three sets of genes: (i) genes found only in a single mycobacterial species, with the other lineage as closest partner; (ii) genes found in more than one mycobacterial species and the other lineage, with no requirement that the other lineage be the closest partner; and (iii) genes found in the mycobacterial species and a close comparator species from the same genus. Genes in set (i) are the best candidates for HGT into the target mycobacterial lineage, while set (ii) will contain many sequences of likely vertical descent. Set (iii) serves as a calibration to set the expected distribution of patristic distances within the genus *Mycobacterium*. In all three examined cases (Figure 5), the mean patristic distance between the target mycobacterial species and its congener was lower than the mean distance for sets (i) and (ii). For the *M. abscessus-Segniliparus* and *M. smegmatis-Streptomyces* pairs, the mean distance of lineage-restricted genes to the other genus was less than that of the non-restricted genes. *M. vanbaalenii*, which had far fewer lineage-restricted genes in trees, showed the opposite effect. While a subset of lineage-restricted proteins may be good candidates for recent HGT, many show divergence times that are more consistent with ancient divergence and loss, or transfers that predate the divergences of at least some of the mycobacterial lineages in our study.

**Figure 6:**
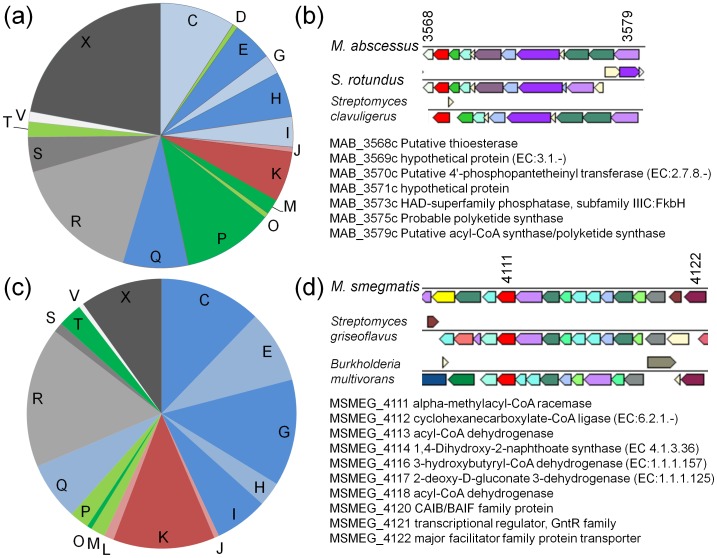
Putative function and genomic context of putative laterally transferred genes. (a, c) distribution of putative transfers according to COG category for pairings of *Segniliparus* with *M. abscessus* (a), and *Streptomyces* with *M. smegmatis* (c). Alternating shades of blue indicate metabolic categories, green indicates cellular processes, red indicates information storage and processing, and hypothetical and unclassified proteins are shown in gray. (b,d) examples of sets of putatively transferred genes showing conserved linkage. Colors indicate different COGs with distinct IDs. Locus names and corresponding gene names are shown, and the numeric portion of the first and last locus IDs is matched to the corresponding locus in the mycobacterial chromosomal segment.

Building on this distributional information, we assessed the functional context of the most-likely transfers involving both the *Segniliparus*-*M. abscessus*, and the *Streptomyces-M. smegmatis* pairings. In each case, we built a set of best transfer candidates by combining two smaller sets: (i) all genes for which the match between the mycobacterial copy to that of the non-mycobacterial partner had a patristic distance ≤66% of the distance between the mycobacterial copy and its homolog in another mycobacterium, and (ii) genes found in the target and no other mycobacterium, whose sister in the phylogenetic tree was the non-mycobacterial target. Set (i) covers potential orthologous replacement events in which a native copy of a gene is replaced, while set (ii) may represent gain of function through HGT. The combination of sets (i) and (ii) for the *Segniliparus*-*M. abscessus* pairing contained a total of 146 genes, with COG category distribution shown in Figure 6a. Apart from a single translation-associated enzyme (a putative formyltransferase), all recovered informational genes (COG categories J, K, and L) are transcription factors, mostly of the TetR family that are typically involved in responses to environmental stress and pathogenicity [40]. Host interactions are suggested by many of the putatively transferred genes, which encode seven polyketide synthases, twelve MmpL/MmpS membrane proteins, and several non-ribosomal peptide synthetases, thioesterases and oligopeptide transporters [41]. Comparison of gene order in *S. rugosus* and *M. abscessus* revealed little in the way of conserved linkage, although some blocks of conserved genes were found. In one case, strong conserved linkage was found for a set of genes likely involved in polyketide synthesis (Figure 6b), with strong conservation between *M. abscessus*, *S. rotundus*, and the more distantly related *Streptomyces clavuligerus*.

The *Streptomyces-M. smegmatis* pairing showed a similar pattern of transferred informational genes, dominated by 27 transcriptional regulators of several families. Perhaps reflecting a primarily non-host-associated lifestyle, putative transfers were relatively enriched in genes encoding metabolic enzymes and transporters, notably carbohydrate transport (COG category G) and energy production (category C). The diversity of regulators was also wider than that observed in the *Segniliparus*-*M. abscessus* pairing above, with nine different families represented including several members of the GntR family which is often associated with carbohydrate utilization [42]. As above, linked sets of putatively transferred genes were in the minority, but a small number of large gene sets with conserved linkage were observed. Most notably, a set of apparent carbohydrate and fatty acid metabolic genes spanning not only *M. smegmatis* and *Streptomyces griseoflavus* but also the proteobacterium *Burkholderia multivorans* was found.

## Discussion

One of the most problematic aspects of reconstructing bacterial evolutionary histories using phylogenetic and comparative genomic analyses is the chance that HGT renders two sequences similar not because the organisms share a recent common ancestor, but because one or both taxa have horizontally acquired a genomic segment from another distantly related species. If concatenated sequences have conflicting evolutionary histories, then a key assumption of phylogenetic tree construction methods is violated, and phylogenetic trees with incorrect topologies and/or misleading branch lengths can be produced [5], [43]–[45].

Detecting recombination events is in itself a complex task, as many, if not most, recombination events do not leave any trace on the recombinant organism’s genome. More specifically, a recombination event could only be detectable if the parental sequences differ at more than two nucleotide positions. Usually, however, such minimal nucleotide differences are not sufficient. Here we used a very conservative approach to test the null hypothesis that recombination has not occurred within the homologous (or core) genomes of 18 mycobacterial species and strains by searching for evidence of recombination events in homologous sequences that had both 1) phylogenetic support and 2) statistical support from at least 3 out of seven different recombination detection methods. Since some of the detected recombinant fragments were small, obtaining statistical support for internal branches of tree topologies was not always possible. Also, including events that were detected by more than three of the methods implemented in RDP3.42 [33], but which did not have phylogenetic support might have yielded an increased rate of false positives (at least for methods like GENECONV, CHIMAERA, 3SEQ and MAXCHI that do not employ phylogenetic information in their recombination detection algorithms). This could have been especially true if the sequences involved in the potential recombination events were evolving under positive selection, where an increase in polymorphisms in some lineages and not others might obscure the correlation between genetic similarity and evolutionary relatedness. On the other hand, gene duplication followed by gene loss, may yield alternative phylogenetic topologies, which may then be confused with HGT [46].

Our analysis of recombination indicated that this evolutionary process most likely only occurs sporadically between core regions of different strains and species of mycobacteria (with a minimum of 9 and a maximum of 19 recombination events detected among homologous sequences depending on the statistical tests employed). The apparent lack of recombination in core regions may in part be reflective of ecological isolation and presumably physical isolation as well: propinquity has been identified as an important requirement for HGT [47]. However, ecological differentiation is not an absolute barrier to HGT among organisms, as recently demonstrated in a study of *Escherichia coli* and *Shigella* [48]; in the case of mycobacteria, broad-host-range mycobacteriophages [49] may serve as vectors to facilitate between-habitat HGT. The recombination detection methods we used are capable of detecting recombination between ancestral mycobacterial lineages that existed prior to ecological differentiation, however such events would be partially obscured by subsequent substitutions [50], leading to diminished statistical significance of inferred events. Our results are also unlikely to be an artifact of sampling, as these methods are generally capable of detecting recent recombination events using datasets containing the recombinant genomes and distant relatives of only one or the other of their parent genomes [51]. The fact that so few homologous recombination events were detected therefore indicates that this form of HGT is unlikely to be a major driver of mycobacterial diversification.

Phylogenomic analyses of 20 mycobacterial strains, incorporating the core genomic regions analyzed for homologous recombination and the “variable” genomic regions including genes found in only a single strain, identified considerable evidence for gene transfer with other members of the Corynebacterinae. Outside of this suborder, certain other genera within the Actinobacteria (notably *Streptomyces*, a group thought to share genes frequently) and to a lesser extent non-Actinobacterial groups (e.g., *Burkholderia*) were frequently found in association with some but not all mycobacterial lineages. Lineages such as *Streptomyces* and *Burkholderia* contain many members with large genomes, and appear frequently as transfer partners with many different groups, often constituting “hubs” in networks of genomes that are linked by HGT [52]. However, as illustrated with the *Segniliparus* here, phylogenetic affinities with a particular group do not necessarily indicate a direct relationship via HGT, and sequencing other microbes from the same habitat may identify much more similar genes. The presence of certain genes in many taxonomically distinct but ecologically linked microbes is consistent with the notion of genes as “public goods” [53], rather than entities that are obligately vertically inherited. Analysis of patristic distances in some cases supported conclusions of recent HGT, but in other cases identified distance distributions more consistent with vertical inheritance and loss from many lineages. The relative lack of highly similar genes in the *M. vanbaalenii*-*Streptomyces* pairing suggests that much of the apparent evidence for frequent HGT may actually be indicative instead of paralogy or gene loss in many other lineages. In defining the gene sets shows in Figure 6 our aim was to analyze a high-confidence set with a low risk of false positives; more distantly related genes include plausible candidates for transfer as well but are likely intermingled with vertically inherited genes as well.

While homologous recombination within the core genome of mycobacteria is likely a relatively rare occurrence, our further analysis of mycobcacterial genomic regions that were found only within specific lineages (i.e. the non-core genome) indicated non-homologous recombination is probably far more pervasive. Less than half of the gene trees that we constructed resolve the mycobacterial genus as a discrete clan suggesting the likelihood of sequence transfers into and out of the genus is fairly high. *M. abscessus* presented with the highest percentage of Group II and IV classified genome fragments, indicating that it is likely the most prolific participant in HGT events of all the mycobacterial species that were considered here. It must, however, be stressed that in many instances our data are also consistent with the hypothesis that various lineage-restricted genome segments could have been present in an ancestral mycobacterial population and were subsequently lost in various species during the course of their diversification. In many cases, further studies involving denser sampling of specific genes within the mycobacteria and their nearest non-mycobacterial relatives would be necessary to definitively demonstrate that HGT and not widespread gene loss accounted for the patterns of phylogenetic relatedness detected here for these genes.

We have shown that both the core genome regions and lineage-restricted genome segments of mycobacteria exhibit pervasive alignment, statistical, and phylogenetic support for recombination events having taken place both amongst individuals within the genus and between mycobacteria and other bacterial species spread throughout the phylum Actinobacteria. Besides illuminating the potential importance of recombination during the evolution of this important group of human pathogens our results indicate that in future molecular evolution studies of mycobacterial sequences, steps should be taken to account for the potentially biasing effects of recombination on any analysis methods that rely on the accurate inference of mycobacterial phylogenetic trees.

## Materials and Methods

### 16S rRNA Gene and Core Genome Tree Construction

Full-length, reference 16S rRNA gene sequences were acquired from the October 2, 2011 update of the GreenGenes server. These sequences were aligned using the NAST protocol as implemented in mothur version 1.16.1 [54], using default parameters. The resulting alignment was subjected to phylogenetic analysis using FastTree version 2.1.0 [55], with a four-category gamma model of among-site rate variation, and a slower, more exhaustive search (parameters: -spr 4 -mlacc 2–slownni).

Mycobacterial genomes used in this work were obtained from NCBI in August 2009. To test the main null hypothesis of clonality, we performed an all-versus-all BLAST comparison of the predicted proteins from the mycobacterial genomes, followed by a greedy aggregation of BLAST matches into conserved homologous groups, each aggregation containing sequences linked by a similarity score of at least 10^−30^ [56], [57]. A total of 6899 homologous groups of sequences were obtained. Each group was further subdivided so that sequences in each set did not differ by more than 30% in their length and the only sets containing a minimum of 4 sequences were considered. A total of 4837 groups satisfied these criteria. Sequences contained in each of the 4837 sets were aligned using Fast Statistical Alignment (FSA) [58]. The 3451 groups containing single-copy gene alignments and were set aside, while the remaining 1386 homologous gene groups were further subdivided into 2980 subsets whose sequences were characterized by at least 70% identity, ignoring gaps (a degree of similarity that renders the sequences alignable). Of the 2980 subdivided homologous sets, 2354 had only single-copy genes, but only 74 of these had ubiquitous single-copy genes. Only homologous sets that contained genes from all 18 species were concatenated in the chromosomal order in which the genes are found in the *M. tuberculosis* F11 reference sequence. This produced a 720,090 nt long sequence alignment. ModelTest [59] analyses indicated that the GTR [60] +G+I models of nucleotide substitution fit the data best [freqA = 0.1630, freqC = 0.3484, freqG = 0.3411, freqT = 0.1475; -lnL = 3913781.7500, proportion of invariable sites (I) = 0.3504, and gamma distribution shape parameter (G) = 1.6071]. We conducted Maximum likelihood (ML) analyses on the concatenated homologous genome segments of the 18 mycobacterial species in RaxML [61] using the best fitting nucleotide substitution model as indicated by the ModelTest analyses [59].

### Recombination Detection, Statistical Tests, and Phylogenetic Analyses

Detection of potential recombinant sequences, identification of likely parental sequences, and localization of potential recombination breakpoints was carried out using RDP 3.42 [33]. RDP3.42 is a comparative program that employs several recombination detection methods: RDP [62], Geneconv [63], MaxChi [64], BootScan [65], 3Seq [66], SiScan [67], and Chimaera [68]. In general, recombination can be detected if one or more descendants of a recombinant have been sampled and at least one sequence resembling a parental sequence has been sampled. Given the variation in sequence length and nucleotide diversity, we performed analyses with a Bonferroni-corrected *p*-value cutoff of <0.05, using default settings except that window sizes were changed to 90 variable nucleotide positions (vnps) for RDP, 400 total nucleotide positions (tnps) for Bootscan, 140 vnps for MaxChi, 120 vnps for Chimaera, and 200 tnps for SiScan [67]. Only those recombination events identified with a P<0.05 statistical support by a minimum of three methods, and for which there was accompanying approximate phylogenetic support (automatically determined during recombination detection by heuristically comparing neighbor joining tree topologies) were further evaluated. ML trees were constructed using PHYML [69] with automated best-fit model selection carried out in RDP3.42.

In cases where, depending on the genome fragment analyzed, the recombinant sequence was situated within different clades of the mycobacterial phylogenetic trees (an obvious signal of recombination having possibly occurred), we used RAxML, TREE-PUZZLE and CONSEL to test for statistically meaningful differences between the topologies of trees using a variety of tests [70], [71]. ML trees of all concatenated homologues both including and excluding recombinant portions, were computed using RAxML [61] and TREE-PUZZLE. We used TREE-PUZZLE with default settings and specified option “-wsl” and we conducted a ML search using RAxML using the gamma model for rate heterogeneity so that the programs would output site-by-site log-likelihoods for each of the statistically significant recombinant portions detected by RDP3.42 [33]. The site-log-likelihood scores file for each tree was subsequently analyzed using the program CONSEL [71]. The statistical tests performed by CONSEL were used to test the null hypothesis that all trees (*i.e.*, ML trees made from the recombinant section and the ML tree made from the concatenated non-recombinant portions of the full *Mycobacterium* 720,090 nt sequence alignment) were equally good explanations, given the available information within the datasets used to construct the trees, of the evolutionary relationships between the sequences within the two trees. When the null hypothesis was rejected (p-value <0.05) for a sub-alignment tree (*i.e*., for each recombinant section of the alignment) vs. full recombination-free alignment/tree comparison, the alternative hypothesis maintaining that the ML tree constructed from the recombinant section is a better fit for the data in the identified recombinant region than the ML tree computed from the concatenated non-recombinant sections, was supported – a result that was taken as definitive phylogenetic confirmation of a recombination event.

### Phylogenomic Analysis of Mycobacterial Proteins

All predicted proteins from the 20 mycobacterial genomes obtained from NCBI were searched against the ‘nr’ database, acquired in October 2011, using version 4.1.93 of USEARCH [72] with a maximum e-value threshold of 10^−30^, a minimum matching length of 70% of the target sequence, and a maximum of ten rejected queries prior to search termination. For each query mycobacterial protein, the bitscore of the best non-self match was identified, and only those results with a bitscore ≥70% of the best score were retained. The resulting sets were used for the initial definition of Groups I–IV based on taxonomic distribution as explained in the main text. Suborder Corynebacterinae, which (apart from *Mycobacterium*) must be absent from Group I, present in Group II, absent from Group III, and present in Group IV, was defined as comprising genera *Corynebacterium*, *Rhodococcus*, *Gordonia*, *Dietzia*, *Nocardia*, *Segniliparus*, and *Tsukamurella*.

Protein sets in Groups II–IV were aligned initially using MUSCLE version 3.7 [73] with arguments “-maxiters 1 -diags -sv -distance1 kbit20_3”. These alignments were used to create hidden Markov models (HMMs) using HMMER 3.0 [74], and then realigned according to the trained HMM. Only sets with at least four sequences, 30 alignment columns, a minimum support in any column of 3.0 and a mean support across all columns of 7.0 were retained in the phylogenetic analysis. Phylogenetic trees of these sets were constructed using FastTree as described above in the 16S rRNA gene analysis. Clans and candidate sister groups in unrooted trees were identified using the Dendropy package [75], as were patristic distances between source and target taxa in phylogenetic trees.

For the two most promising lineage pairings (*Segniliparus* with *M. abscessus* and *Streptomyces* with *M. smegmatis*), we constructed high-confidence sets of HGT candidates by combining lineage-restricted genes with genes whose distributions were unrestricted, but had closest affinity to the partner lineage (see Results for details). The resulting gene sets were profiled according to COG functional category and conserved linkage using the Joint Genome Institute Integrated Microbial Genomes system [76].

## Supporting Information

Table S1 **Genomes of Mycobacterium used in this study, including identified habitats.**
(DOC)

Table S2 **Summary of the Statistical Tests Performed by CONSEL.**
(DOC)

Table S3 **Recombination events identified through analysis with RDP3.2.**
(DOC)
