# Supplementary material for: Comparative Genomic and Phylogenetic Approaches to Characterize the Role of Genetic Recombination in Mycobacterial Evolution
Source: PLoS One. 2012 Nov 26;7(11):e50070. doi: 10.1371/journal.pone.0050070 (PMC3506542; doi:10.1371/journal.pone.0050070)
Supplement: Table S1 — Genomes of Mycobacterium used in this study, including identified habitats. (DOC) [file pone.0050070.s001.doc]

Table S1. Genomes of *Mycobacterium* used in this study, including identified habitats.

| Organism name | NCBI accessions | Habitats | genes | Ref |
| --- | --- | --- | --- | --- |
| M. abscessus ATCC 19977 | NC_010397, NC_010394 | Water, opportunistic human pathogen | 4941 | (S1) |
| M. avium 104 | NC_008595 | Host-associated (human); freshwater; soil | 5124 | (S2) |
| M. avium subsp. paratuberculosis K-10 | NC_002944 | Host-associated (bovine) | 4350 | (S3) |
| M. bovis BCG str. Pasteur 1173P2 | NC_008769 | Host-associated (bovine) | 3954 | (S4) |
| M. bovis BCG str. Tokyo 172 | NC_012207 | Host-associated (bovine) | 3944 | (S5) |
| M. gilvum PYR-GCK | NC_009339, NC_009340, NC_009341, NC_009338 | Soil | 5580 | (S6) |
| M. leprae Br4923 | NC_011896 | Host-associated (human) | 1604 | (S7) |
| M. leprae TN | NC_002677 | Host-associated (human) | 1606 | (S8) |
| M. marinum M | NC_010604, NC_010612 | Host-associated (fish, amphibian, human skin); freshwater | 5452 | (S9) |
| M. smegmatis str. MC2 155 | NC_008596 | Soil, water, opportunistic human pathogen | 6716 | (S10) |
| Mycobacterium sp. JLS | NC_009077 | Soil | 5739 | (S11) |
| Mycobacterium sp. KMS | NC_008703, NC_008704, NC_008705 | Soil | 5975 | (S12) |
| Mycobacterium sp. MCS | NC_008147, NC_008146 | Soil | 5615 | (S13) |
| M. tuberculosis CDC1551 | NC_002755 | Host-associated (human) | 4189 | (S14) |
| M. tuberculosis F11 | NC_009565 | Host-associated (human) | 3942 | (S15) |
| M. tuberculosis H37Ra | NC_009525 | Host-associated (human) | 4034 | (S16) |
| M. tuberculosis H37Rv | NC_000962 | Host-associated (human) | 3989 | (S17) |
| M. tuberculosis KZN 1435 | NC_012943 | Host-associated (human) | 4059 | (S15) |
| M. ulcerans Agy99 | NC_005916, NC_008611 | Pathogenic, host-associated (human); freshwater | 4241 | (S18) |
| M. vanbaalenii PYR-1 | NC_008726 | Sediment; soil | 5980 | (S19) |

References for Table S1

(S1) Ripoll F, Pasek S, Schenowitz C, Dossat C, Barbe V, Rottman M, Macheras E, Heym B, Herrmann JL, Daffé M, Brosch R, Risler JL, Gaillard JL. 2009. Non mycobacterial virulence genes in the genome of the emerging pathogen Mycobacterium abscessus. PLoS ONE 4:e5660.

(S2) Semret M, Zhai G, Mostowy S, Cleto C, Alexander D, Cangelosi G, Cousins D, Collins DM, van Soolingen D, Behr MA. 2004. Extensive genomic polymorphism within Mycobacterium avium. J Bacteriol 186:6332-4.

(S3) Li L, Bannantine JP, Zhang Q, Amonsin A, May BJ, Alt D, Banerji N, Kanjilal S, Kapur V. 2005. The complete genome sequence of Mycobacterium avium subspecies paratuberculosis. Proc Natl Acad Sci USA 102:12344-9.

(S4) Brosch R, Gordon SV, Garnier T, Eiglmeier K, Frigui W, Valenti P, Dos Santos S, Duthoy S, Lacroix C, Garcia-Pelayo C, Inwald JK, Golby P, Garcia JN, Hewinson RG, Behr MA, Quail MA, Churcher C, Barrell BG, Parkhill J, Cole ST. Proc Natl Acad Sci USA 104:5596-601.

(S5) Seki M, Honda I, Fujita I, Yano I, Yamamoto S, Koyama A. 2009. Whole genome sequence analysis of Mycobacterium bovis bacillus Calmette-Guérin (BCG) Tokyo 172: a comparative study of BCG vaccine substrains. Vaccine 27:1710-1716.

(S6) unpublished (see <http://www.ncbi.nlm.nih.gov/genome/?term=txid350054>)

(S7) Monot M, Honoré N, Garnier T, Zidane N, Sherafi D, Paniz-Mondolfi A, Matsuoka M, Taylor GM, Donoghue HD, Bouwman A, Mays S, Watson C, Lockwood D, Khamesipour A, Dowlati Y, Jianping S, Rea TH, Vera-Cabrera L, Stefani MM, Banu S, Macdonald M, Sapkota BR, Spencer JS, Thomas J, Harshman K, Singh P, Busso P, Gattiker A, Rougemont J, Brennan PJ, Cole ST. 2009. Comparative genomic and phylogeographic analysis of Mycobacterium leprae. Nat Genet 41:1282-9.

(S8) Cole ST, Eiglmeier K, Parkhill J, James KD, Thomson NR, Wheeler PR, Honoré N, Garnier T, Churcher C, Harris D, Mungall K, Basham D, Brown D, Chillingworth T, Connor R, Davies RM, Devlin K, Duthoy S, Feltwell T, Fraser A, Hamlin N, Holroyd S, Hornsby T, Jagels K, Lacroix C, Maclean J, Moule S, Murphy L, Oliver K, Quail MA, Rajandream MA, Rutherford KM, Rutter S, Seeger K, Simon S, Simmonds M, Skelton J, Squares R, Squares S, Stevens K, Taylor K, Whitehead S, Woodward JR, Barrell BG. 2001. Massive gene decay in the leprosy bacillus. Nature 409: 1007-11.

(S9) Stinear TP, Seemann T, Harrison PF, Jenkin GA, Davies JK, Johnson PD, Abdellah Z, Arrowsmith C, Chillingworth T, Churcher C, Clarke K, Cronin A, Davis P, Goodhead I, Holroyd N, Jagels K, Lord A, Moule S, Mungall K, Norbertczak H, Quail MA, Rabbinowitsch E, Walker D, White B, Whitehead S, Small PL, Brosch R, Ramakrishnan L, Fischbach MA, Parkhill J, Cole ST. 2008. Insights from the complete genome sequence of Mycobacterium marinum on the evolution of Mycobacterium tuberculosis. Genome Res. 18:729-41.

(S10) Unpublished (see <http://www.ncbi.nlm.nih.gov/sites/entrez?db=bioproject&cmd=Retrieve&list_uids=92>)

(S11) Unpublished (see <http://www.ncbi.nlm.nih.gov/genome/13563?project_id=58489>)

(S12) Unpublished (see <http://www.ncbi.nlm.nih.gov/genome/13563?project_id=58491>)

(S13) Unpublished (see <http://www.ncbi.nlm.nih.gov/genome/13563?project_id=58465>)

(S14) Fleischmann RD, Alland D, Eisen JA, Carpenter L, White O, Peterson J, DeBoy R, Dodson R, Gwinn M, Haft D, Hickey E, Kolonay JF, Nelson WC, Umayam LA, Ermolaeva M, Salzberg SL, Delcher A, Utterback T, Weidman J, Khouri H, Gill J, Mikula A, Bishai W, Jacobs Jr WR Jr, Venter JC, Fraser CM. 2002. Whole-genome comparison of Mycobacterium tuberculosis clinical and laboratory strains. J Bacteriol 184:5479-90.

(S15) Unpublished (see http://www.broadinstitute.org/annotation/genome/mycobacterium_tuberculosis_spp/MultiHome.html)

(S16) Zheng H, Lu L, Wang B, Pu S, Zhang X, Zhu G, Shi W, Zhang L, Wang H, Wang S, Zhao G, Zhang Y. 2008. Genetic basis of virulence attenuation revealed by comparative genomic analysis of Mycobacterium tuberculosis strain H37Ra versus H37Rv. *PLoS ONE* 3:e2375.

(S17) Cole ST, Brosch R, Parkhill J, Garnier T, Churcher C, Harris D, Gordon SV, Eiglmeier K, Gas S, Barry CE 3rd, Tekaia F, Badcock K, Basham D, Brown D, Chillingworth T, Connor R, Davies R, Devlin K, Feltwell T, Gentles S, Hamlin N, Holroyd S, Hornsby T, Jagels K, Krogh A, McLean J, Moule S, Murphy L, Oliver K, Osborne J, Quail MA, Rajandream MA, Rogers J, Rutter S, Seeger K, Skelton J, Squares R, Squares S, Sulston JE, Taylor K, Whitehead S, Barrell BG. 1998. Deciphering the biology of Mycobacterium tuberculosis from the complete genome sequence. Nature 393:537-44.

(S18) Stinear TP, Seemann T, Pidot S, Frigui W, Reysset G, Garnier T, Meurice G, Simon D, Bouchier C, Ma L, Tichit M, Porter JL, Ryan J, Johnson PD, Davies JK, Jenkin GA, Small PL, Jones LM, Tekaia F, Laval F, Daffé M, Parkhill J, Cole ST. 2007. Reductive evolution and niche adaptation inferred from the genome of Mycobacterium ulcerans, the causative agent of Buruli ulcer. Genome Res 17:192-200.

(S19) Kim SJ, Kweon O, Jones RC, Edmondson RD, Cerniglia CE. 2008. Genomic analysis of polycyclic aromatic hydrocarbon degradation in Mycobacterium vanbaalenii PYR-1. Biodegradation 19:859-81.
