# Supplementary material for: Comparative Genomic and Phylogenetic Approaches to Characterize the Role of Genetic Recombination in Mycobacterial Evolution
Source: PLoS One. 2012 Nov 26;7(11):e50070. doi: 10.1371/journal.pone.0050070 (PMC3506542; doi:10.1371/journal.pone.0050070)
Supplement: Table S2 — Summary of the Statistical Tests Performed by CONSEL. (DOC) [file pone.0050070.s002.doc]

**Table S2**. Summary of the Statistical Tests Performed by CONSEL.

| **Tree #** | **RDP Event #** | **AU Test** | **SE** | **SH Test** | **SE** | **WSH test** | **SE** | **H_0_ Rejected?** | **For which tests?** | **Gene/protein involved** |
| --- | --- | --- | --- | --- | --- | --- | --- | --- | --- | --- |
| 1 | 1 | 2.00E-4 | 0.001 | 0.000 | 0.000 | 0.000 | 0.000 | yes | all | NADPH-dependent FMN reductase |
| 2 | 2 | 3.00E-35 | 0.000 | 0.000 | 0.000 | 0.000 | 0.000 | yes | all | NADPH-dependent FMN reductase |
| 3 | 3 | 5.00E-7 | 0.000 | 0.000 | 0.000 | 0.000 | 0.000 | yes | all | NADPH-dependent FMN reductase |
| 4 | 4 | 3.00E-4 | 0.002 | 0.033 | 0.002 | 0.000 | 0.000 | yes | all | F0F1 ATP synthase subunit beta/delta |
| 5 | 5 | 9.00E-64 | 0.000 | 0.003 | 0.001 | 0.000 | 0.000 | yes | all | oxidoreductase |
| 6 | 6 | 3.00E-29 | 0.000 | 0.000 | 0.000 | 0.000 | 0.000 | yes | all | transposase IS116/IS110/IS902 family protein |
| 7 | 7 | 0.024 | 0.005 | 0.334 | 0.005 | 0.115 | 0.003 | yes | for AU only | alpha/beta hydrolase superfamily |
| 8 | 8 | 0.003 | 0.001 | 0.715 | 0.005 | 0.033 | 0.002 | yes | for AU and WSH only | fatty acid synthase |

Table S2: 1) tree number (trees from 28 potential recombination events identified by RDP3.42 (Heath *et al*. 2006), with two identical trees having been merged into one). 2) corresponding RDP3.42 event number. 3) The p-value for the AU, SH, and WSH test followed by their respective standard error (SE). 4) null hypothesis testing and the supporting tests. 5) Function of the protein region involved in the potential recombination event.

Calculations made with CONSEL (Shimodaira and Hasegawa, 2001).

| AU = p-value of approximately unbiased test | | | |  |
| --- | --- | --- | --- | --- |
| SE= standard error |  | |  | |
| SH= Shimodaira-Hasegawa test | |  | | |
| WSH= weighted Shimodaira-Hasegawa | | | |  |

Table S2 continued

| **Tree #** | **RDP Event #** | **AU Test** | **SE** | **SH Test** | **SE** | **WSH test** | **SE** | **H_0_ Rejected?** | **For which tests?** | **Gene/protein involved** |
| --- | --- | --- | --- | --- | --- | --- | --- | --- | --- | --- |
| 9 | 9 | 5.00E-60 | 0.000 | 0.000 | 0.000 | 0.000 | 0.000 | yes | all | NADPH-dependent FMN reductase |
| 10 | 10 | 0.01 | 0.004 | 0.234 | 0.004 | 0.082 | 0.003 | yes | for AU test only | FeS assembly protein SufB |
| 11 | 11 | 0.223 | 0.011 | 0.760 | 0.004 | 0.588 | 0.005 | no |  | beta-ketoacyl synthase; acyl transferase domain protein; MaoC domain protein dehydratase; 2-nitropropane dioxygenase, NPD; domain of unknown function |
| 12 | 12 | 0.153 | 0.011 | 0.705 | 0.005 | 0.599 | 0.005 | no |  | succinyl-CoA synthase beta chain |
| 13 | 13 | 2.00E-70 | 0.000 | 0.089 | 0.003 | 2.00E-004 | 0.000 | yes | for AU and WSH only | phosphatidate cytidylyltransferase |
| 14 | 16 | 2.00E-10 | 0.000 | 0.104 | 0.003 | 0.003 | 0.001 | yes | for AU and WSH only | fructose 1,6-bisphosphatase II |
| 15 | 19 | 4.00E-47 | 0.000 | 0.001 | 0.000 | 0.000 | 0.000 | yes | all | gene:dnaG; protein product: DNA primase |
| 16 | 22 | 0.612 | 0.110 | 0.985 | 1.000 | 0.971 | 0.002 | no |  | gene:cdd; protein product: cytidine deaminase |
| 17 | 24 | 0.141 | 0.010 | 0.767 | 0.004 | 0.496 | 0.005 | no |  | molecular chaperone DnaK; grpE Hsp-70 cofactor; |

Table S2 continued

| **Tree #** | **RDP Event #** | **AU Test** | **SE** | **SH Test** | **SE** | **WSH test** | **SE** | **H_0_ Rejected?** | **For which tests?** | **Gene/protein involved** |
| --- | --- | --- | --- | --- | --- | --- | --- | --- | --- | --- |
| 18 | 27 | 0.059 | 0.008 | 0.675 | 0.005 | 0.278 | 0.004 | maybe | for AU only | grpE Hsp70 cofactor; dnaK 70 kD heat shock protein |
| 19 | 28 | 4.00E-5 | 0.000 | 0.267 | 0.004 | 0.001 | 0.000 | yes | for AU and WSH only | enoyl-(acyl carrier protein) reductase |
| 20 | 31 | 0.782 | 0.008 | 0.996 | 0.001 | 0.997 | 0.001 | no |  | ketol-acid reductoisomerase ilvC |
| 21 | 33 | 0.002 | 0.001 | 0.224 | 0.004 | 0.025 | 0.002 | yes | for AU and WSH only | DNA Topoisomerase (TOP4). |
| 22 | 34 | 0.002 | 0.001 | 0.602 | 0.005 | 0.108 | 0.003 | yes | for AU and WSH only | ATP synthase subunit epsilon |
| 23 | 37 | 3.00E-4 | 0.001 | 0.028 | 0.002 | 0.001 | 0.000 | yes | all | Pks14 |
| 24 | 39 | 0.053 | 0.013 | 0.512 | 0.005 | 0.383 | 0.005 | maybe | for AU only | succinyl-CoA synthetase subunit alpha |
| 25 | 45 | 0.717 | 0.008 | 0.977 | 0.001 | 0.978 | 0.001 | no |  | ruvA Holliday junction DNA helicase RuvA |
| 26 | 48 | 0.397 | 0.020 | 0.960 | 0.002 | 0.939 | 0.002 | no |  | inorganic pyrophosphatase ppa |
| 27 | 62 | 0.346 | 0.014 | 0.642 | 0.005 | 0.852 | 0.004 | no |  | undecaprenyldiphospho-muramoylpentapeptide beta-N- acetylglucosaminyltransferase; UDP-N-acetylmuramate--L-alanine ligase; putative cell division protein FtsQ |
| 28 | 74 | 0.002 | 0.002 | 0.220 | 0.004 | 0.002 | 0.000 | yes | for AU and WSH only | Protein region: DoxX |
