# Supplementary material for: Comparative Genomic and Phylogenetic Approaches to Characterize the Role of Genetic Recombination in Mycobacterial Evolution
Source: PLoS One. 2012 Nov 26;7(11):e50070. doi: 10.1371/journal.pone.0050070 (PMC3506542; doi:10.1371/journal.pone.0050070)
Supplement: Table S3 — Recombination events identified through analysis with RDP3.2. (DOC) [file pone.0050070.s003.doc]

**Table S3.** Recombination events identified through analysis with RDP3.2.

| **Recomb. Event #**  **(in RDP File)** | **Recombinant Sequence(s)** | **Minor Parental Sequence(s)** | **RDP** | **GENECONV** | **Bootscan** | **Maxchi** | **Chimaera** | **Si**  **Scan** | **Phyl**  **Pro** | **LARD** | **3**  **Seq** |
| --- | --- | --- | --- | --- | --- | --- | --- | --- | --- | --- | --- |
| 1 (1) | M_sp_JLS | M_ulcerans  M_tb_H37Rv  M_tb_H37Ra  M_marinum  M_avium_paratb  M_avium_22579  M_bovis_BCG  M_tb_F11  M_bovis_17071  M_tb_1551 | 2.1E-160 | 7.22E-49 | NS | 4.26E-16 | 290R-049 | NS | NS | NS | NS |
| 2 (2) | M_ulcerans | M_gilvum  M_vanbaalenii | 1.39E-32 | NS | NS | NS | 2.66E-003 | NS | NS | NS | NS |
| 3 (3) | M_leprae | M_abscessus | 4.49E-30 | 2.17E-07 | NS | 4.86E-09 | 8.45E-12 | NS | NS | NS | NS |
| 4 (4) | M_smegmatis | M_vanbaalenii | 2E-23 | NS | NS | 1.54E-003 | 2.6E-09 | NS | NS | NS | NS |
| 5 (5) | M_a_paratb  M_a_22579 | M_abscessus | 1.07E-20 | NS | NS | 1.44E-08 | 5.52E-10 | NS | NS | NS | NS |

Table S3: This table details the recombinant and potential parental sequence(s) of the RDP events identified in S1. The events highlighted in yellow are the 9 events with the highest degree of statistical support.

Table S3 continued

| **Recomb. Event #**  **(in RDP File)** | **Recombinant Sequence(s)** | **Minor Parental Sequence(s)** | **RDP** | **GENECONV** | **Bootscan** | **Maxchi** | **Chimaera** | **Si**  **Scan** | **Phyl**  **Pro** | **LARD** | **3**  **Seq** |
| --- | --- | --- | --- | --- | --- | --- | --- | --- | --- | --- | --- |
| 6 (6) | M_vanbaalenii  M_smegmatis | M_abscessus | 1.02E-19 | NS | NS | 0.000432 | 0.000247 | NS | NS | NS | NS |
| 7 (7) | M_gilvum | Unknown (M_sp_MCS)  Unknown (M_sp_JLS)  Unknown (M_sp_KMS) | 3.64E-19 | NS | NS | 1.13E-004 | 1.88E-05 | NS | NS | NS | NS |
| 8 (8) | M_sp_MCS  M_sp_JLS  M_sp_KMS  M_smegmatis | M_marinum  M_tb_H37Rv  M_tb_H37Ra  M_bovis_BCG  M_tb_F11  M_bovis_17071  M_ulcerans | 5.7E-13 | NS | NS | 2.22E-05 | 1.02E-06 | NS | NS | NS | NS |
| 9 (9) | M_gilvum  M_vanbaalenii | M_avium_paratb  M_avium_22579 | 9.02E-11 | NS | 1.01E-09 | 7.74E-004 | 4.92E-002 | NS | NS | NS | NS |
| 10 (10) | M_gilvum | M_sp_KMS  M_sp_JLS  M_sp_MCS | 3.07E-10 | NS | 1.3E-09 | 1.7E-05 | 3.88E-05 | NS | NS | NS | NS |
| 11 (11) | M_vanbaalenii | M_smegmatis | 2.08E-09 | NS | 1.08E-08 | 6.73E-004 | 2.87E-05 | NS | NS | NS | NS |

Table S3 continued

| **Recomb. Event #**  **(in RDP File)** | **Recombinant Sequence(s)** | **Minor Parental Sequence(s)** | **RDP** | **GENECONV** | **Bootscan** | **Maxchi** | **Chimaera** | **Si**  **Scan** | **Phyl**  **Pro** | **LARD** | **3**  **Seq** |
| --- | --- | --- | --- | --- | --- | --- | --- | --- | --- | --- | --- |
| 12 (12) | M_leprae  M_tb_H37Rv  M_tb_H37Ra  M_bovis_BCG  M_tb_F11  M_bovis_17071  M_tb_1551 | Unknown (M_abscessus)  Unknown (M_smegmatis) | 3.51E-09 | NS | 1.86E-09 | 2.41E-06 | 4.72E-10 | NS | NS | NS | NS |
| 13 (13) | M_gilvum | M_sp_MCS  M_sp_JLS  M_sp_KMS | 1.95E-08 | NS | 3.83E-08 | NS | 0.015478 | NS | NS | NS | NS |
| 14 (16) | M_gilvum | M_smegmatis | 5.45E-07 | NS | 4.27E-07 | 2.54E-003 | 3.34E-05 | NS | NS | NS | NS |
| 15 (19) | M_leprae | Unknown (M_abscessus)  Unknown (M_smegmatis) | 4.09E-05 | 2.6E-10 | NS | NS | NS | NS | NS | NS | NS |
| 16 (22) | M_leprae | Unknown (M_vanbaalenii)  Unknown (M_sp_JLS)  Unknown (M_abscessus)  Unknown (M_sp_MCS)  Unknown (M_sp_KMS)  Unknown (M_smegmatis) | 7.45E-06 | NS | NS | 0.000506 | 1.6E-05 | NS | NS | NS | NS |

Table S3 continued

| **Recomb. Event #**  **(in RDP File)** | **Recombinant Sequence(s)** | **Minor Parental Sequence(s)** | **RDP** | **GENECONV** | **Bootscan** | **Maxchi** | **Chimaera** | **Si**  **Scan** | **Phyl**  **Pro** | **LARD** | **3**  **Seq** |
| --- | --- | --- | --- | --- | --- | --- | --- | --- | --- | --- | --- |
| 17 (24) | M_tb_H37Rv  M_tb_H37Ra  M_bovis_BCG  M_tb_F11  M_bovis_17071  M_tb_1551 | Unknown (M_vanbaalenii) | 9.74E-06 | NS | NS | NS | 0.023026 | NS | NS | NS | NS |
| 18 (27) | M_leprae | Unknown (M_vanbaalenii) | 1.57E-05 | NS | NS | 2.18E-05 | 0.048099 | NS | NS | NS | NS |
| 19 (28) | M_smegmatis | M_vanbaalenii | 2.51E-05 | NS | NS | 0.0454 | 0.000268 | NS | NS | NS | NS |
| 20 (31) | M_tb_H37Rv  M_tb_H37Ra  M_bovis_BCG  M_tb_F11  M_bovis_17071  M_tb_1551 |  | 3.69E-05 | NS | NS | NS | 0.007013 | NS | NS | NS | NS |
| 21 (33) | M_abscessus |  | 0.000115 | NS | NS | NS | 0.021362 | NS | NS | NS | NS |
| 22 (34) | M_smegmatis |  | 0.000146 | NS | NS | NS | 0.036576 | NS | NS | NS | NS |
| 23 (37) | M_avium  M_a_paratb | M_leprae | 0.000283 | 0.001182 | NS | 0.004128 | NS | NS | NS | NS | NS |
| 24 (39) | M_marinum  M_ulcerans | M_bovis_BCG  M_tb_H37Rv  M_tb_H37Ra  M_tb_F11  M_bovis_17071  M_tb_1551 | 0.000441 | NS | NS | 9.56E-07 | 1.53E-06 | NS | NS | NS | 3.03E-004 |

Table S3 continued

| **Recomb. Event #**  **(in RDP File)** | **Recombinant Sequence(s)** | **Minor Parental Sequence(s)** | **RDP** | **GENECONV** | **Bootscan** | **Maxchi** | **Chimaera** | **Si**  **Scan** | **Phyl**  **Pro** | **LARD** | **3**  **Seq** |
| --- | --- | --- | --- | --- | --- | --- | --- | --- | --- | --- | --- |
| 25 (45) | M_gilvum  M_vanbaalenii | Unknown (M_leprae) | 0.00113 | NS | NS | 2.94E-002 | NS | NS | NS | NS | NS |
| 26 (48) | M_tb_H37Ra  M_tb_H37Rv  M_bovis_BCG  M_tb_F11  M_bovis_17071  M_tb_1551 | Unknown (M_vanbaalenii) | 0.001828 | NS | NS | NS | 3.47E-003 | NS | NS | NS | NS |
| 27 (62) | M_abscessus | M_sp_JLS  M_sp_MCS  M_sp_KMS | 0.008869 | 0.011652 | NS | NS | NS | NS | NS | NS | NS |
| 28 (74) | M_leprae | M_avium_paratb  M_avium_22579 | 0.027056 | NS | NS | NS | 4.38E-003 | NS | NS | NS | NS |
